# Circadian Adaptation to Night Shift Work Influences Sleep, Performance, Mood and the Autonomic Modulation of the Heart

**DOI:** 10.1371/journal.pone.0070813

**Published:** 2013-07-26

**Authors:** Philippe Boudreau, Guy A. Dumont, Diane B. Boivin

**Affiliations:** 1 Centre for Study and Treatment of Circadian Rhythms, Douglas Mental Health University Institute, Department of Psychiatry, McGill University, Montreal, Quebec, Canada; 2 Integrated Program in Neuroscience, McGill University, Montreal, Quebec, Canada; 3 Department of Electrical and Computer Engineering, University of British Colombia, Vancouver, British Colombia, Canada; Pennsylvania State University, United States of America

## Abstract

Our aim was to investigate how circadian adaptation to night shift work affects psychomotor performance, sleep, subjective alertness and mood, melatonin levels, and heart rate variability (HRV). Fifteen healthy police officers on patrol working rotating shifts participated to a bright light intervention study with 2 participants studied under two conditions. The participants entered the laboratory for 48 h before and after a series of 7 consecutive night shifts in the field. The nighttime and daytime sleep periods were scheduled during the first and second laboratory visit, respectively. The subjects were considered “adapted” to night shifts if their peak salivary melatonin occurred during their daytime sleep period during the second visit. The sleep duration and quality were comparable between laboratory visits in the adapted group, whereas they were reduced during visit 2 in the non-adapted group. Reaction speed was higher at the end of the waking period during the second laboratory visit in the adapted compared to the non-adapted group. Sleep onset latency (SOL) and subjective mood levels were significantly reduced and the LF∶HF ratio during daytime sleep was significantly increased in the non-adapted group compared to the adapted group. Circadian adaptation to night shift work led to better performance, alertness and mood levels, longer daytime sleep, and lower sympathetic dominance during daytime sleep. These results suggest that the degree of circadian adaptation to night shift work is associated to different health indices. Longitudinal studies are required to investigate long-term clinical implications of circadian misalignment to atypical work schedules.

## Introduction

According to recent American and European surveys, between 15 and 30% of the adult population is involved in some type of shift work, with 19% of the European population reportedly working at least 2 h between 22:00 h and 05:00 h [1], [2]. Night work has been associated with reduced sleep duration [3]–[8], typically ranging from 4–7 h, symptoms of insomnia during the main sleep period [9], and sleepiness across wake periods [5], [10]–[12]. Slower performance at work [10], [13]–[18], especially when measured during the first night shift [19]–[21], has also been reported. These sleep and cognitive deficits may lead to enhanced injury risk in working environments. According to the 2010 National Health Interview Survey, less than 6 h of sleep per night is associated with an 86% increased risk in work-related injury compared to 7–8 h of sleep [22]. During night shifts, police officers have a 72% increased risk of injury compared to day shifts [23], thus leading to inflated injury-compensation claims in the shift work population [24].

Most shift workers experience a disruption in the temporal alignment between endogenous circadian rhythms and their atypical sleep-wake schedule. In most individuals, the circadian pacemaker does not rapidly adapt to phase shifts, as evidenced by a lack of entrainment of the core body temperature, melatonin, and cortisol rhythms to a night schedule [3], [13], [25]–[27]. This state of chronic circadian misalignment can lead to sleep and performance complaints but also contributes to the association between night work and adverse health outcomes, such as elevated risk of cardiovascular diseases [28], [29], metabolic syndrome [29], diabetes [29], autoimmune hypothyroidism [30], and specific cancer types [29], [31], [32] when compared to the general population. We recently showed an interaction between the sleep-wake and circadian processes regulating heart rate, leading to altered autonomous nervous system (ANS) modulation of the heart when sleep occurs at adverse circadian phases [33], [34]. These results suggest that the state of circadian alignment to shift work could contribute to cardiovascular health.

Individuals exposed to shift work greatly vary in their capacity to adapt to their shifted schedule [14], [17], [18], [35]. Some shift workers may intuitively adopt behaviors that favor circadian re-entrainment, while others, either because of domestic responsibilities, inappropriate sleep hygiene or inconsistent light/dark exposure, will never adapt to the point that some may develop shift work sleep disorder [36]. We previously reported that bright light therapy can be used to help nurses [3], [26] and healthy participants [25] to entrain to night work. However, our recent intervention in police officers on patrol showed a much more variable circadian adaptation to 7 consecutive night shifts, presumably due to the suboptimal use of bright light therapy [13]. In the present study, we reanalyzed this previous dataset to investigate how circadian adjustment to night shift work affects the hormone levels, sleep, psychomotor performance, subjective alertness and mood, as well as heart rate variability (HRV) of police officers on patrol.

## Methods

### Participants

Fifteen police officers on patrol (7 men and 8 women; mean age ± SD: 30.1±5.2 years) were recruited. Participants provided written informed consent prior to their participation in this study. All experimental procedures were approved by the Douglas Mental Health University Institute Research Ethics Board and are within the ethical standards of the Declaration of Helsinki. Two of the men participated in both conditions, namely bright light therapy and control conditions, for a total of 17 experimental laboratory evaluations. Their work shift schedule followed a predetermined 35-day rotating shift sequence (3 evening shifts, 2 rest days, 4 day shifts, 2 rest days, 7 night shifts, 6 rest days, 4 evening shifts, 2 rest days, 3 day shifts, and 2 rest days), with the night shifts starting between 22:00 to 23:30 h and lasting between 8 to 8.5 h. The screening procedures consisted of medical and psychological questionnaires and examinations, as well as routine blood and urine analyses. The exclusion criteria included sleep disorders, intrinsic circadian rhythm disorders and illicit drug use (i.e., phencyclidine, benzodiazepines, cocaine, amphetamines, tetrahydrocannabinol, opiates, and barbiturates; Triage Drugs of Abuse Panel, Biosite, San Diego, CA, USA). Their demographic data were reported in a prior publication [13]. The chronotype was measured with the Horne & Ostberg Morningness-Eveningness Questionnaire (MEQ) [37].

Five to seven days prior to the first laboratory admission, the officers lived on a day-oriented schedule, working either day shifts or evening shifts, or being on rest days. They also maintained a regular 8-h nocturnal sleep period and avoided daytime napping. Time in and out of bed was verified by daily phone calls to the laboratory, a sleep-wake log, and actigraphic recordings (AW-64, Mini Mitter-Respirotronics, Bend, OR, USA).

### Procedures

**Figure 1:**
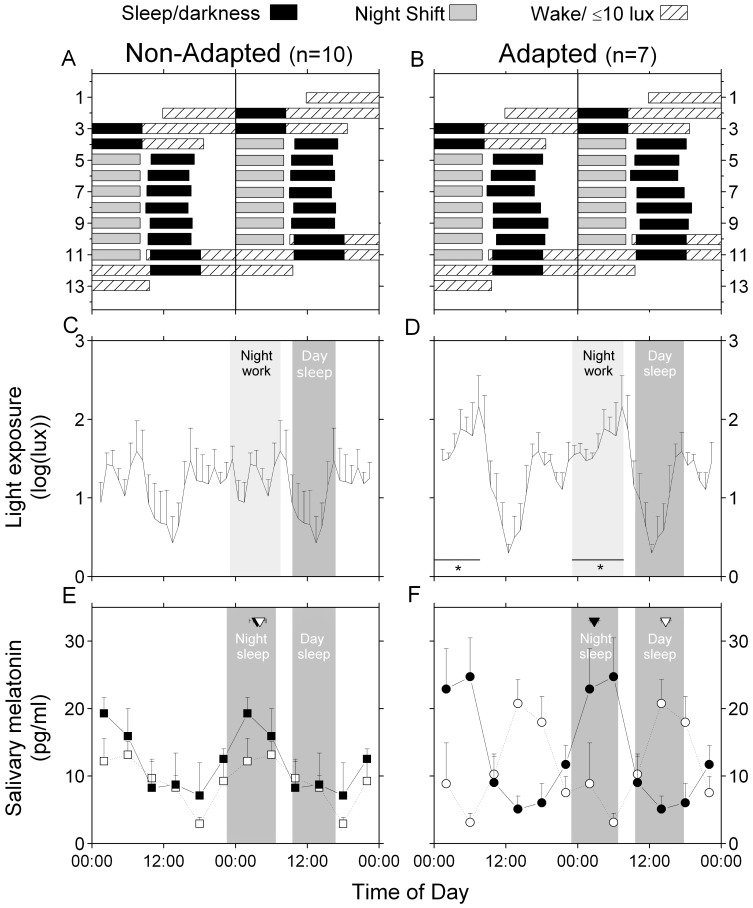
Experimental protocol, light exposure, and mean expression of salivary melatonin during both laboratory visits. The panels on the left show the results of the non-adapted group, whereas the panels on the right show the results of the adapted group. The upper panels (A and B) show the experimental protocol and the average sleep times for each adaptation group. The sleep/darkness periods are indicated by black bars, the wake periods in the laboratory are indicated by hatched bars, and the work shifts are indicated by grey bars. Panels C and D illustrate the mean 24-h pattern of light exposure (log transformed) during the ambulatory period. For each adaptation group, the mean night shift work period is illustrated by a light gray box, and the mean day sleep period is illustrated by a dark gray box. Asterisks (*) linked with a horizontal line indicate significantly greater levels of light exposure (p<0.05) in the adapted group compared to the non-adapted group during the night shift. Panels E and F illustrate the mean salivary melatonin rhythm in the non-adapted group (square symbols) and adapted group (circle symbols) during laboratory visit 1 (filled symbols, solid lines) and laboratory visit 2 (open symbols, dashed lines). For each adaptation group, the mean day and night sleep periods in the laboratory are illustrated by dark gray boxes. The mean acrophases were averaged across the adaptation groups and laboratory visits and are illustrated by inverted triangles. The mean melatonin level was averaged across adaptation groups for each laboratory visit based on clock time. To facilitate visualization, all panels are double plotted, and in panels C to F, the night shift work and sleep periods were only added for the second 24 hours. All values are means ± SEM.

The officers were studied in the laboratory for 48 consecutive h, before and after the series of 7 consecutive night shifts as part of their 35-day schedule. The experimental procedure is illustrated in Figure 1 (panels A & B).

During the first laboratory visit, two 8-h nocturnal sleep periods were scheduled according to the officers' average bed- and wake-time (i.e., nighttime sleep) as determined by their sleep-wake log 5–7 days prior to laboratory admission. As all participants followed the same 35-day work roster schedule, they were on 1 evening shift, 2 rest days, and 4 day shifts the week prior to admission. During the second visit, two 8-h sleep periods were scheduled to start 2 h after the end of their last night shift (i.e., daytime sleep). The sleep periods were scheduled in total darkness. During the wake periods in the laboratory, the participants were requested to maintain low levels of activity and the light intensities within the laboratory suite were kept very dim (<10 lux). During both laboratory visits, the participants were served breakfast, lunch, dinner, and a snack at 30 min, 6 h, 12 h, and 14 h after the scheduled time of awakening, respectively. Coffee or caffeinated products were not allowed in the laboratory. Coffee and tea consumption during the ambulatory period were the same in the adapted (mean ± SD: 0.8±1.0 cup/day) and non-adapted groups (0.7±1.0 cup/day), and HRV and PSG recordings were performed at least 24 h after the last caffeinated beverage was consumed.

#### Adaptation groups

The present study is a reanalysis of data that were previously published on the efficacy of a light/darkness intervention of police officers working night shifts [13]. In this initial study, the police officers were randomly assigned to one of two study groups: control (n = 9) or intervention (n = 8). Two officers participated in both conditions one year apart. The order of study for these two participants was counterbalanced between the subjects. Here, we grouped data from the police officers based on their level of circadian adaptation to night shift. The participants were considered adapted to night shift work if their salivary melatonin acrophase occurred during the daytime sleep period of their second visit. They were considered non-adapted otherwise. Seven officers (4i, 5c, 5i, 6c, 7i, 11c, 15c; thus 3 out of 8 in the intervention “i” group, and 4 out of 9 in the control “c” group) were classified as adapted to their night schedule, whereas 10 were not (1c, 1i, 2i, 3c, 8c, 9c, 10c, 12i, 13i, 16i; thus, 5 out of 8 in the intervention group and 5 out of 9 in the control group).

### Measurements and data processing

During the 7 consecutive night shifts, the light levels were monitored by a light sensor worn at the collar of the officer's jacket (Micro Light Sensor, Ambulatory Monitoring, NY, USA). The sensitivity of this device range from 0 to 4000 lux. The participants were instructed to wear the light sensor from awakening to bedtime and to place it on their bedside desk during their sleep periods. The police officers in the intervention group also had to wear orange-tinted goggles from sunrise to bedtime or if they woke up during or before the end of their daytime sleep period [13]. Light exposure measurements were corrected when the police officers of the intervention group were wearing orange-tinted goggles by using a transmission coefficient of 48%. Because our assessment of light exposure did not quantify the spectral composition of the environmental light (irradiance) but measured illuminance (in lux), we propose this transmission estimate by considering 1) an estimated outdoor light spectrum (light color temperature of 5000 K, Figure S2, [38]), 2) the transmission filter of the orange-tinted goggles (Figure S1 and S2) [39], and 3) the luminous function to transform radiant energy into luminous energy [40] (see Methods S1 for details). We validated this estimate experimentally by measuring the environmental illuminance with and without the orange-tinted goggles in Montreal in the morning with a research photometer (IL1400A, International Light, Peabody, MA, USA). When we correct for the known spectral sensitivity of the human circadian system to light [41], we estimate that these goggles block approximately 96.7% of the effects of environmental light on the circadian system (Figure S3). The participants also reported bedtime, time out of bed, and naps in a sleep-wake log during their night shifts. Sleep periods at home following each night shift were considered in the ambulatory sleep analyses. These analyses excluded the 7^th^ daytime sleep period because it was scheduled to occur in the laboratory.

During each laboratory visit, saliva samples were collected every 60 min during the wake periods and every 2 h during the first sleep period by gently waking up the participants without turning the lights on. The technicians used a flashlight directed away from the participant's eyes to collect samples during the sleep period. To collect the saliva samples, the officers were asked to spit in a test tube or to use salivettes (Sarstedt, Montréal, Canada). The saliva samples were assayed in duplicate for their melatonin content. Details on the melatonin assay kit have been previously published [13]. At each laboratory visit, for each adaptation group, the salivary melatonin levels were binned every 4 h to illustrate a circadian profile of secretion.

Every sleep period in the laboratory was recorded polysomnographically (PSG) on a computerized system (Harmonie, Natus Medical Inc., Montreal, Qc, Canada). The PSG recordings included a central and occipital electroencephalogram (EEG), an electrooculogram (EOG), and a submental electromyogram (EMG). All channels were sampled at 250 Hz. High- and low-pass filters were applied to the EEG (0.3 Hz and 35 Hz), EMG (5 Hz and 35 Hz), EOG (0.1 Hz and 15 Hz) and EKG (1 Hz and 35 Hz) signals. The PSG sleep recordings were visually scored according to standard criteria using 30-sec epochs [42]. The following parameters were calculated for each sleep period. Time in bed (TIB) was the time between lights-out and lights-on. Sleep onset latency (SOL) was defined as the time interval between lights out and the first occurrence of at least two consecutive epochs of stage 1 (S1) sleep or any occurrence of a deeper sleep stage. REM sleep onset latency (ROL) was the time interval between sleep onset and the first occurrence of an epoch of REM sleep. Sleep period (SP) was defined as the time between sleep onset and the final awakening. NREM sleep was the amount of time spent in stage 1 to 4 (S1, S2, S3, S4) sleep during the SP, and SWS was the amount of time spent in S3 and S4 sleep. Total sleep time (TST) was defined as the time spent in NREM sleep and REM sleep. Sleep efficiency (SE) was calculated as TST/TIB×100%. Wake after sleep onset (WASO) was the amount of time spent awake during the SP. Periodic leg movements in sleep (PLMs) and sleep apneas/hypopneas were ruled out during the first nocturnal sleep period. For PLMs, EMGs of the left and right anterior tibialis were recorded. Leg movements occurring at intervals of 4.0–90.0 sec and clustered in groups of ≥4 were considered PLMs, in accordance with Coleman's criteria [43]. Respiratory parameters were monitored with a bucconasal thermistor and an airflow pressure transducer. The AASM recommended criteria [44] for defining sleep apnea (≥90% reduction in airflow for ≥10 sec) and hypopnea (≥30% reduction in airflow for ≥10 sec) were used.

Ten-minute psychomotor vigilance task (PVT) sessions were planned every 2 h in the laboratory during wake periods, for a total of 14 tests per laboratory visit. For each PVT session, the median reaction speed (1/reaction time) and the 10% fastest and slowest reaction speeds were calculated. Subjective alertness and mood were assessed every 20 min using 10-cm visual analog scales, ranging from 0 cm (sleepy or sad) to 10 cm (alert or happy). No difference in the absolute levels of subjective alertness and mood scores was observed between adaptation groups during visit 1. Thus, subjective alertness and mood scores at both visits were transformed using z-scores that were calculated based on the individual baseline mean and standard error during the first laboratory visit. Physical and psychological workload, and stress related to work were measured using 10-cm visual analog scales (low to high) after each work shift.

EKG was continuously recorded (250 Hz) during sleep periods using PSG recordings. R-peaks were extracted from the EKG signal using automatic detection software (VivoLogic, Vivometrics, Ventura, CA, USA). RR interval (RRI) data were visually inspected for ectopic beats and artifacts and were manually corrected by linear interpolation. The spectral power of the RRI signal was calculated by a discrete wavelet transform (DWT) using an *ad hoc* Matlab program (Matlab 7.4, MathWorks, Natick, MA, USA) and the Wavelab toolbox [45] (see [33] for the detailed DWT analysis). The HRV was described by the spectral power in 2 standard frequency bands (i.e., high frequency power [HF] and low frequency power [LF]) [46]. High frequencies (HF; 0.15 to 0.4 Hz) reflect the parasympathetic modulation of the heart, whereas low frequencies (LF; 0.04 to 0.15 Hz) reflect the ANS modulation of the heart as a whole. The LF/HF ratio is often used to monitor the sympathovagal balance. The wavelet coefficients that best matched each of these recommended frequency bands were selected (HF: 0.15–0.30 Hz; LF: 0.0375–0.15 Hz). Prior to the statistical analysis, the HRV data were collapsed into 30-sec bins to correspond to the 30-sec PSG epochs.

### Data and statistical analyses

Ambulatory light levels were log transformed (log(x+1)), averaged into 1-h bins, and folded every 24 h for illustrative purposes. The light exposure of each subject was averaged across 4 different periods of the 24-h day: the night shift, morning commute home, daytime sleep, and the evening prior to the next shift. These periods were based on each participant's schedule (see Table S1). The light during the morning commute was defined as the time between the end of the night shift and the beginning of the daytime sleep period, while the evening light exposure was defined as the time between the end of the daytime sleep period and the beginning of the night shift. The salivary melatonin acrophase of each subject was calculated using a triple harmonic regression [47]. The light levels (factors: adaptation group×time of day), melatonin acrophase (factors: adaptation group×visit), and measurements collected with the sleep-wake log (factor: adaptation group) were then compared using a mixed linear model (*mixed* SAS procedure, SAS Institute Inc., Cary, NC, USA). This statistical procedure took into consideration the 2 subjects that participated in the study twice (i.e., both in the intervention and control groups).

The PSG parameters and HRV data collected during the sleep periods in the laboratory were compared between adaptation groups, laboratory visits, and sleep stages (only for HRV) using the *mixed* SAS procedure. Only the data during the second sleep period of each laboratory visit was analyzed because the officers were woken during the first sleep period of each laboratory visit to collect saliva samples. For the HRV analysis, only the data collected during the wake epochs during the TIB were included.

A mixed linear model was also applied to the PVT data, subjective alertness and mood scores that were collected in the laboratory using the *mixed* SAS procedure. The factors used for these analyses included adaptation group, laboratory visit, and number of hours awake. In all of the analyses, the significance level was set at p<0.05, and the main effects and interactions were further investigated using the differences between the least squares means with Tukey's correction for multiple comparisons. The results are expressed as the means ± SEM unless otherwise mentioned. All data were checked for normality.

## Results

The adapted and non-adapted groups were similar in terms of the proportion of participants that were initially assigned to the control or intervention conditions (Chi-square test, p = 0.95). The PSG and HRV data of subject 2i were excluded from the analysis because the PSG recording of the first laboratory visit was missing, and thus, no within-visit comparison was possible for this subject. The participants in the adapted group were significantly younger (mean age ± SD: 26.4±5.1 years) than those in the non-adapted group (32.6±5.3 years; p = 0.011). There was a trend for participants of the adapted group to be slightly more evening types than the non-adapted participants (MEQ score: 42.1±8.4 vs. 51.9±8.6, respectively; p = 0.055). Physical and psychological workload and stress related to work were similar between the adaptation groups. No difference was observed between the adaptation groups, on results of a 10-cm visual analog scale (calm/excited) administered 3×/hour during wake periods in the laboratory.

### Ambulatory sleep-wake behavior

Prior to laboratory visit 1, no difference in bedtime or waketime was observed between the adaptation groups (p≥0.36) based on their sleep-wake logs. During this period, a trend was observed for TIB to be reduced in the adapted group compared to the non-adapted group (adapted: 7.77±0.23 h; non-adapted: 8.16±0.08 h; p = 0.084). During the week of ambulatory night shifts, a trend was observed for the adapted group to get out of bed later than the non-adapted group (adapted: 17:48±00:16 h; non-adapted: 16:42±00:26 h; p = 0.074). No significant difference was observed in the average bedtime (adapted: 09:38±00:11 h; non-adapted: 09:32±00:30 h; p = 0.88) or nap length (adapted: 5.4±7.2 min/day; non-adapted: 13.1±7.2 min/day; p = 0.41) or in the variability of bedtime or time out of bed between the adaptation groups (p≥0.45).

### Ambulatory light exposure

The average 24-h light levels during the ambulatory night shifts are presented in Figure 1 (panels C & D). The ambulatory light exposure values from subjects 1c, 1i, and 4i were excluded from this analysis because the light sensors were lost (1c and 4i) or because of technical problems during the recording period (1i). The start time and duration of the 4 time periods during which the light levels were averaged are reported in Table S1. No significant difference was observed between the adaptation groups, except for a trend of a shorter duration of the “evening prior to the next night shift” period in the adapted group (p = 0.078). This observation is consistent with the trend towards longer daytime sleep duration observed in the adapted group. A significant adaptation group×time-of-day interaction was observed in light exposure (p = 0.043). More specifically, higher light levels were observed for the adapted group compared to the non-adapted group during their night shifts (p = 0.047). No other difference in light exposure was observed between the adaptation groups (p≥0.23).

### Hormone data

The mean salivary melatonin profile of each adaptation group during both laboratory visits are illustrated in Figure 1 (panels E & F). More details about the individual salivary melatonin rhythms can be found in our prior publication [13]. A significant adaptation group×visit interaction was observed for the timing of the melatonin acrophase (p = 0.0002). The salivary melatonin acrophase was significantly delayed by −11.95±2.4 h in the adapted group between laboratory visits (acrophase visit 1: 02:46±0:41 h; visit 2: 14:43±0:39 h; p<0.001), whereas it was delayed by −0.44±1.7 h in the non-adapted group (acrophase visit 1: 03:37±1:20 h; visit 2: 04:03±1:07 h; p = 0.77). The salivary melatonin acrophase was also significantly delayed during visit 2 in the adapted group compared to the non-adapted group (p<0.001), while no difference was observed during visit 1 (p = 0.61).

### PSG recordings

**Figure 2:**
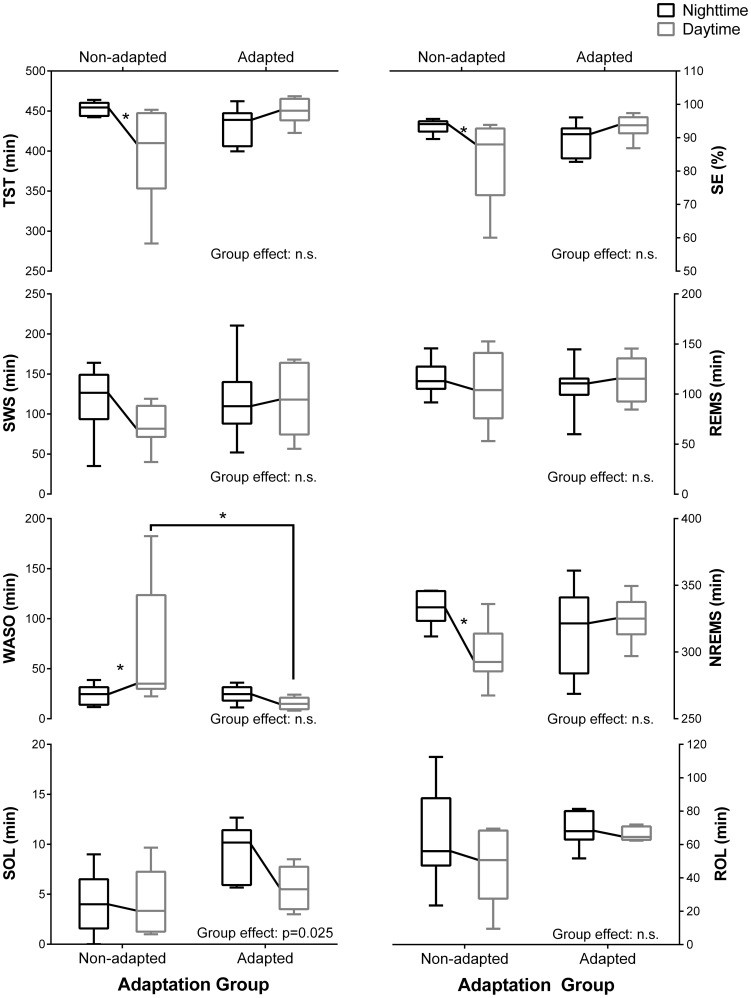
Variation in the PSG sleep measurements during nighttime and daytime sleep (laboratory visits 1 and 2) in both adaptation groups. Nighttime (black boxes) and daytime (grey boxes) sleep measurements are illustrated by boxplots. The bottom and top of each box are the first and third quartiles of each group, respectively, and the band inside each box is the median. The top and bottom whiskers illustrate the 5th and 95th percentile, respectively, for each group. TST: total sleep time; SE: sleep efficiency; SWS: slow wave sleep; REMS: rapid eye movement sleep; WASO: wake after sleep onset; NREMS: non-REM sleep; SOL: sleep onset latency; ROL: REMS onset latency. The PSG sleep measurements were compared using a linear mixed effect model with visit and adaptation group as factors. Asterisks (*) indicates a significant difference (p<0.05) between laboratory visits or between groups.

The details of the PSG recordings are reported in Table 1 and Figure 2. A significant main effect of adaptation group was observed for the SOL (p = 0.025), with shorter latency in the non-adapted group. A significant visit×adaptation group interaction was observed for the TST (p = 0.005), SE (p = 0.008), WASO (p = 0.016), and NREM sleep (p = 0.015). With the exception of WASO that was reduced during daytime compared to night time sleep, no difference was observed in the adapted group between visits. This indicates similar high quality sleep between the daytime and nighttime sleep periods. In comparison, a significant increase in the WASO value (p≤0.047) and reduction in the TST, SE and NREM sleep values (p≤0.049) was observed during daytime sleep compared to nighttime sleep in the non-adapted group. Finally, WASO was lower (P≤0.048) and there was a trend for higher TST (p = 0.069) in the adapted group compared to the non-adapted group during the second laboratory visit.

**Table 1:** PSG recordings during nighttime and daytime sleep in the adapted and non-adapted group.

	Non-Adapted		Adapted				
	Nighttime (V1)	Daytime (V2)	Nighttime (V1)	Daytime (V2)	Visit Effect	Group	Interaction
Bedtime	23.06±0.16	9.55±0.21	22.86±0.38	9.96±0.19	n.s.	n.s.	n.s.
Waketime	7.06±0.11	17.52±0.23	6.85±0.41	17.98±0.18	n.s.	n.s.	n.s.
TST (min)	453.2±2.94	398.52±19.31†	431.48±8.79	449.36±5.89	n.s.	n.s.	p = 0.005
SE (%)	0.93±0.01	0.83±0.04†	0.9±0.02	0.93±0.01	n.s.	n.s.	p = 0.008
SOL (min)	4.24±1	4.13±1.06	9.28±1.15	5.61±0.89	P = 0.029	p = 0.025	n.s.
ROL (min)	64.37±9.23	48.17±7.65	68.64±3.84	66.37±1.89	n.s.	n.s.	n.s.
WASO (min)	23.94±3.32	71.52±19.1*†	24.5±3.56	15.28±2.49*†	n.s.	n.s.	p = 0.016
WASO (%)	5.31±0.76	15.02±4.50*†	5.69±0.92	4.83±0.57*†	n.s.	n.s.	p = 0.030
S1 (min)	3.94±0.93	6.57±1.45	4.47±0.99	8.26±2.44	n.s.	n.s.	n.s.
S1 (%)	0.87±0.18	1.71±0.4	1.04±0.24	1.87±0.56	p = 0.034	n.s.	n.s.
S2 (min)	216.19±16.6	198.89±13.36	199.05±22.78	206.79±19.55	n.s.	n.s.	n.s.
S2 (%)	48.78±3.51	50.1±3.31	46.04±4.87	46.18±4.59	n.s.	n.s.	n.s.
SWS (min)	117.04±14.45	87±8.39	117.79±18.97	117.88±16.08	n.s.	n.s.	n.s.
SWS (%)	25.57±2.81	21.82±1.83	27.29±4.23	26.1±3.39	n.s.	n.s.	n.s.
REMS (min)	116.04±5.84	106.06±11.78	106.1±9.57	116.43±8.33	n.s.	n.s.	n.s.
REMS (%)	24.79±1.31	26.37±2.3	24.59±2.28	25.86±1.7	n.s.	n.s.	n.s.
NREMS (min)	333.23±4.44	298.31±7.56†	316.83±12.33	324.67±6.36	n.s.	n.s.	p = 0.020
NREMS (%)	74.35±1.29	71.92±2.22	73.33±1.87	72.28±1.33	n.s.	n.s.	n.s.

Details of the PSG recording values during the nighttime (visit 1 [V1]) and daytime (visit 2 [V2]) sleep periods in the adapted and non-adapted groups. TST: total sleep time; SE: sleep efficiency; SOL: sleep onset latency; REMS: rapid eye movement sleep; ROL: REMS latency; NREMS: non-REM sleep; WASO: wake after sleep onset; S1: stage 1 sleep; S2: stage 2 sleep; SWS: slow wave sleep. The PSG measurements were compared using a linear mixed effect model with visit and adaptation group as factors and the results are reported in the last three columns.

† indicate significant differences (p<0.05) between laboratory visits (thus between nighttime and daytime sleep).

Asterisks (*) indicate significant differences (p<0.05) between adaptation groups. All values are means ± SEM.

### PVT analysis

#### Mean reaction speed

**Figure 3:**
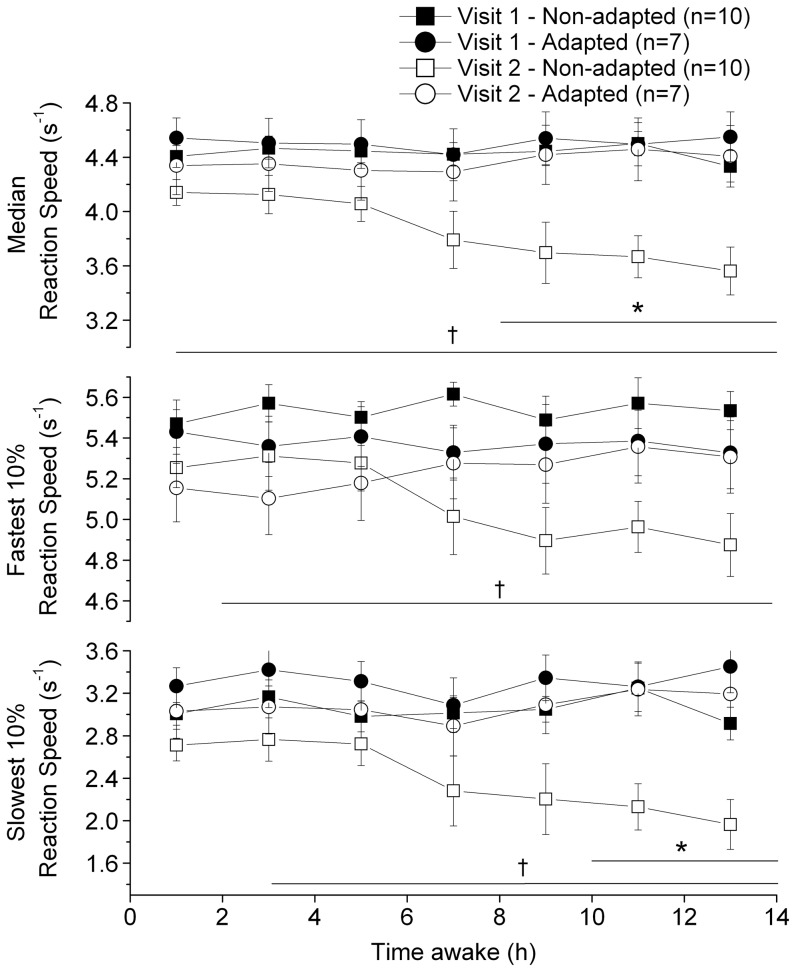
PVT performances as a function of time awake during laboratory visits 1 and 2 in both adaptation groups. The results of the non-adapted group (square symbols) and adapted group (circle symbols) obtained during laboratory visit 1 (filled symbols) and laboratory visit 2 (open symbols) are illustrated. The top, middle, and bottom panels illustrate the median reaction speed, fastest 10% reaction speed, and slowest 10% reaction speed, respectively. A linear mixed effect model with time awake, laboratory visit and adaptation group as factors was used to compare the PVT measurements. The time periods during which this model revealed significant differences are illustrated by horizontal lines. Asterisks (*) linked with a horizontal line indicate slower reaction speed (p<0.05) in the non-adapted group compared to the adapted group during laboratory visit 2. † linked with a horizontal line indicate a significant reduction (p<0.05) in the performance measurements between laboratory visits in the non-adapted group. No such difference was observed in the adapted group. For illustrative purposes, the reaction speeds were binned every 2 h and the results are illustrated as means ± SEM at mid-bin.

No main effect of adaptation group (p = 0.88) was observed for the median reaction speed. However, a significant main effect of visit was observed (p = 0.012), with slower reaction speeds during visit 2 compared to visit 1 (Figure 3). A main effect of time awake (p = 0.036) was also observed, with a small increase in reaction time (thus, lower reaction speed) with time spent awake. The 3-way interaction (factors: adaptation group×visit×time awake) was significant (P = 0.046). More specifically, no significant difference was observed between the visits for reaction speed in the adapted group, while the reaction speed was slower following daytime sleep compared to nighttime sleep during the first 14 hours awake in the non-adapted group (p≤0.005). During visit 2, the adapted group was significantly faster than the non-adapted group after 8 to 14 hours awake (p≤0.045).

#### Fastest 10% reaction speed

No main effect of adaptation group (p = 0.25) or time awake (p = 0.30) was observed for the fastest 10% reaction speed. However, a significant main effect of visit was observed (p = 0.006), with a slower reaction speed during visit 2 compared to visit 1 (Figure 3). The 3-way interaction (factor: adaptation group×visit×time awake) was significant (p = 0.012). In the adapted group, no difference was observed between the laboratory visits, whereas in the non-adapted group, the fastest 10% reaction speed was reduced during visit 2 compared to visit 1 after 2 to 14 hours awake (p≤0.02). During the second visit, no difference was observed between the adapted and non-adapted group.

#### Slowest 10% reaction speed

No main effect of adaptation group (p = 0.97) was observed for the slowest 10% reaction speed. However, a significant main effect of visit was observed (p = 0.043), with a slower reaction speed during visit 2 compared to visit 1 (Figure 3). A trend for a main effect of time awake (p = 0.08) was observed, with a small drop in reaction speed with the amount of time spent awake. The 3-way interaction was significant (p = 0.037). More specifically, no significant differences in reaction speed was observed for the adapted group, whereas the reaction speed of the non-adapted group was slower during the second compared to the first visit after 3 to 14 hours awake (p<0.001). During visit 2, the adapted group was significantly faster than the non-adapted group after 10 to 14 hours awake (P≤0.024).

### Subjective alertness

**Figure 4:**
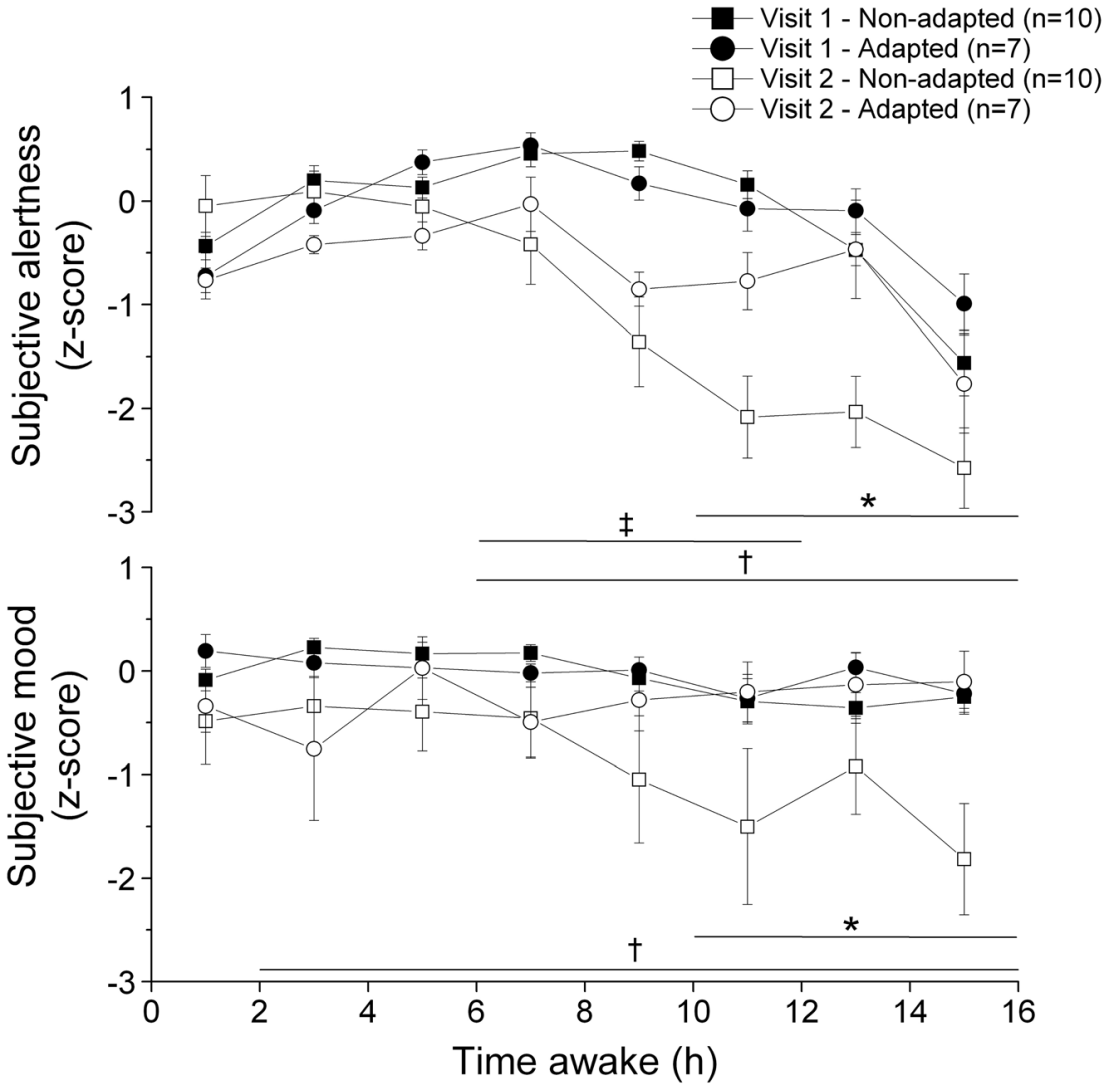
Subjective alertness (upper panel) and subjective mood (lower panel) levels with time awake during laboratory visits 1 (filled symbols) and 2 (open symbols) in the adapted (circles) and non-adapted (squares) groups. Higher values indicate higher scores. A linear mixed effect model with time awake, sleep time and adaptation group as factors was used to compare the subjective alertness and mood levels. The time periods during which this model revealed significant differences are illustrated by horizontal lines. Asterisks (*) linked with a horizontal line indicate lower alertness or mood levels in the non-adapted group compared to the adapted group during laboratory visit 2. † linked with a horizontal line indicate significant differences (p<0.05) in subjective alertness or mood between laboratory visits in the non-adapted group. ‡ linked with and a horizontal line indicates a significant difference between visits in the adapted group. For illustrative purposes, the alertness and mood z-scores were binned every 2 h and the results are illustrated as means ± SEM at mid-bin.

Absolute alertness levels were similar between groups at visit 1 (non-adapted: 7.2±0.5 cm; adapted: 6.7±0.3 cm; p = 0.47). This value was used in the calculation of z-scores. A significant main effect of adaptation group was observed for subjective alertness (p = 0.026, Figure 4), with the adapted group reporting higher alertness levels. The interaction of adaptation group×visit×time awake was also significant (p = 0.005). More specifically, during visit 2, the adapted group reported higher alertness after 10 to 16 h awake compared to the non-adapted group (p≤0.01). In the adapted group, the participants reported lower subjective alertness during visit 2 compared to visit 1 after 6 to 12 h awake (p≤0.04). In the non-adapted group, the participants reported lower subjective alertness during visit 2 compared to visit 1 after 6 to 16 h awake (p<0.001).

### Subjective mood

Absolute mood levels were similar between groups at visit 1 (non-adapted: 1.1±0.4 cm; adapted: 1.7±0.7 cm; p = 0.41). This value was used in the calculation of z-scores. No significant main effect of adaptation group was observed for the subjective mood levels (p = 0.54, Figure 4). However, the interaction of adaptation group×visit×time awake was significant (p<0.001). More specifically, during visit 2, the adapted group reported higher mood scores after 10 to 16 h awake compared to the non-adapted group. While no significant mood difference was observed between visits in the adapted group (p≥0.10), the participants in the non-adapted group reported lower subjective mood levels during visit 2 compared to visit 1 after 2 to 16 h awake (p≤0.02).

### HRV analysis

**Figure 5:**
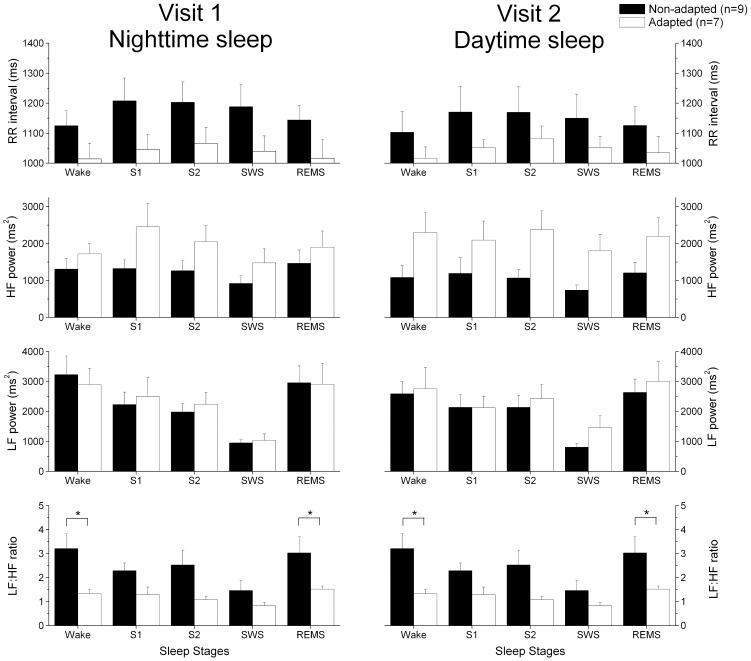
Variation in the consecutive RR intervals, HF power, LF power and LF∶HF ratio during nighttime (visit 1) and daytime (visit 2) sleep. Wake: wake from lights out to lights on; S1: stage 1 sleep; S2: stage 2 sleep; SWS: slow wave sleep; REMS: rapid eye movement sleep. A linear mixed effect model was applied to the data for each subject using the *mixed* SAS procedure. We used 3 comparison factors: adaptation group, laboratory visit, and sleep stages. A significant main effect of sleep stage was observed for all HRV parameters (p≤0.004). There was a trend for the HF power to be increased in the adapted group compared to the non-adapted group (p = 0.07). A significant adaptation group×sleep stages interaction was also observed (p = 0.01). Asterisks (*) indicate a significant differences between the adaptation groups (p<0.05). All values are means ± SEM.

A significant main effect of sleep stage was observed for every HRV parameter analyzed (p≤0.008, Figure 5), with the LF power value and the LF∶HF ratio decreasing and the RR interval increasing with NREM sleep depth compared to wakefulness. The HF power was significantly reduced during SWS compared to stages 1 and 2 (p≤0.03). There was a trend for the HF power to be increased (p = 0.06) and the LF∶HF ratio to be decreased (p = 0.08) in the adapted group compared to the non-adapted group. A significant adaptation group×sleep stage interaction was observed for the LF∶HF ratio. More specifically, the LF∶HF ratio was significantly increased by approximately 2-fold in the non-adapted group compared to the adapted group during wakefulness (+104%, p = 0.015) and REM sleep (+81%, p = 0.049). A trend for a similar increase was observed during stage 2 sleep (+109%, p = 0.06). No other main effect or interaction was observed for the HRV parameters.

## Discussion

We observed circadian adaptation of the salivary melatonin rhythm in 7 out of 17 police officers after 7 consecutive night shifts. The circadian rhythm of salivary melatonin was inverted (−11.95 h) in the adapted group after 7 night shifts, whereas the acrophase was relatively unchanged (−0.44 h) in the non-adapted group. These circadian phase delays are similar to our previous findings in a simulated night shift work experiment [25] and in nurses working at night [26] . Namely, delays observed in our adapted and non-adapted group are similar to those reported in where treatment and control groups of our prior studies, respectively. The group of adapted officers in the present study was comprised of 3 out of 8 and 4 out of 9 officers who were originally assigned to our intervention and control groups, respectively [13]. We discovered that, as a group, the police officers in our original intervention group did not expose themselves to light at night as much as requested [13]. The present study revealed higher light levels at night in the adapted group compared to the non-adapted group, regardless of the original intervention group. These results indicate that the workers' behavior in terms of exposure to light can influence their level of circadian adaptation to night shift work. Consistent with our result, Dumont et al. [48] showed higher light exposure between 04:00 h to 06:00 h in night shift workers who had significant delayed urinary 6-sulphatoxymelatonin rhythm compared to those who did not. Shift workers can achieve total or partial entrainment to their work schedule by judicious use of bright light therapy [14], [17], [18], [49]–[52], as we previously showed using a similar protocol in nurses working permanent night shifts [3].

With no specific recommendation other than sleeping in total darkness, 4 out of 9 (44%) of the police officers in the original control group were adapted to their nighttime schedule after 7 consecutive night shifts. This percentage is much higher than the 2.6% “complete” and the 23.7% combined “complete” and “substantial” spontaneous circadian adaptation to night work that was estimated by Folkard, based on the results of 6 prior studies [53]. Selection bias could partially explain our findings, as highly motivated healthy police officers were recruited. Sleeping in total darkness could also have contributed to circadian adaptation in the control group, especially because the officers already had good regular sleeping habits. Simply by maintaining a regular daily schedule of sleep/darkness exposure for 3 days compared to a free sleep schedule, night shift workers can achieve significant circadian phase delay (∼2.1 h vs. ∼0.48 h), as measured by dim light melatonin onset [54]. Our results suggest that many officers instinctively adopted behaviors that facilitate circadian adaptation in terms of light exposure. When examining the light exposure behavior in the sub-group of adapted officers who were initially assigned to the control group [13], we observed that they also exposed themselves to higher light levels in the late night/early morning (from ∼03:00 h to 07:00 h), and to lower light levels during the daytime sleep period (from ∼10:00 h AM to 15:00 h) compared to the non-adapted officers in the control group. Although the sample size was too small to run reliable statistical analyses for this sub-group, this finding provides further support to the importance of light/darkness exposure for circadian adaptation to night shift work. Spontaneous circadian entrainment to night work has been shown in different populations, including offshore fleet workers [20], oil rig workers [21] and in simulated night shift experiments [19]. The circadian adaptation of these individuals and our police officers was most likely a result of their optimal exposure to environmental synchronizers, such as a favorable light/darkness exposure and the maintenance of a fixed daytime sleep/wake schedule for 7 consecutive days [55].

The officers in the adapted group tended to be more evening types compared to the non-adapted group. To our knowledge, we are the first to report the association between an objective measure of circadian adaptation to night shift work and chronotype. Later bedtimes and wake times on rest days between night shifts [56] and prior to simulated night shifts [14] were associated with a greater circadian phase delay with respect to dim light melatonin onset. Other studies also found that eveningness can contribute to perceived adaptation to night shift work [57], [58].

The average TST and SE values during daytime sleep were similar to those achieved during nighttime sleep in the adapted group. In comparison, the TST and SE were significantly reduced in the non-adapted group by 40.44±14.0 min and 7.2±3.2% when the officers slept during the day compared to at night. A reduced in NREMS duration was observed in non-adapted officers for daytime sleep. This is explained by the non-significant reduction in S2 sleep (−8%) and SWS (−26%) during daytime compared to nighttime sleep. This contrasts with similar amount of S2 sleep and SWS during daytime and nighttime sleep periods in the adapted officers. The results observed in the non-adapted officers are consistent with the reduced TST observed in prior studies reporting subjective [7] and objective [4], [5], [8], [14], [59] measures of sleep in shift workers. The TST was reduced by 72 min (6.7 h vs. 8.6 h) in miners [4] and by 104 min (4.6 h vs. 6.3 h) in nurses [59] working at night compared to their days off. We previously reported that bright light-induced circadian adaptation to night shift work could increase daytime sleep duration from 6.6 h to 7.1 h in nurses who had been working permanent night shifts for 11.5±8.0 years (mean ± SD) [3]. Our past and present findings are consistent with those of Crowley et al. [14], who reported that a longer TST was positively correlated with the degree of circadian adaptation in a simulated night work experiment.

When sleep restriction accumulates over consecutive working days, large sleep deficits may cause acute or chronic fatigue and sleepiness, especially if sufficient time is not allowed for recovery [11]. Even restricting the TIB to 7 h per night for 7 consecutive days, results in a significant 23.6% reduction in psychomotor performance after compared to that resulting from 9 h of daily sleep opportunity [60]. This observation has practical implications considering that 58.9% of the North American population reports sleeping for 7 h or less per night [61]. In our study, the non-adapted officers had average SOLs of 4.24±1.00 min and 4.13±1.06 min when they went to bed at night and during the day, respectively. This situation is consistent with severe and persistent sleepiness (SOL of <5) based on multiple sleep latency test criteria [62] and is similar to what has been observed in patients suffering from shift work disorder [35]. Recent studies have shown that there is great inter-individual variability in the performance and sleepiness response to sleep restriction [63], [64] and shift work [65]. Our results suggest that the officers in the non-adapted group could indeed be more vulnerable to the effects of shift work.

In the non-adapted group, the reaction speeds were significantly slower during the second laboratory visit compared to the first visit, especially after longer time awake at night. In comparison, the psychomotor performance was stable across both visits in the adapted group. These results are consistent with studies examining the impact of night shift work [14]–[18] and sleep restriction [10], [11], [60], [63] on performance [66]. In a simulated night shift experiment, Lamond et al. [67] reported that during the last shift of 7 consecutive nights, the reaction speed decreased by 0.75% per hour awake, while we found a performance impairment of almost twice this size (−1.39%/h) in our non-adapted group and stable performance with time awake in our adapted group (∼0%/h) during the second laboratory visit. This difference can be explained by an average 5.5 h circadian phase delay in the study of Lamond et al. [19], [67], a phase shift that is approximately between those that we observed in our adaptation groups. This finding emphasizes the usefulness of assessing circadian phase in night work experiments. As it was the case for TST, psychomotor performance has been correlated with circadian phase delay in night shift workers [14], [17]. Total [19] or partial [17], [18] circadian re-entrainment by phase delay shifts can produce substantial performance improvement in night shift workers, especially in young subjects [14]. Circadian adaptation to night shift work could thus be advantageous with respect to safety.

The subjective mood and alertness levels were significantly lower after 10 h awake in the non-adapted group compared to the adapted group during the second laboratory visit. This result is consistent with the drop in alertness levels in the early morning reported in long haul drivers [68], night nurses [69] and during simulated night shift work [14], [18]. Subjective alertness was also reduced in the adapted group after 6 to 12 h awake during the second compared to the first laboratory visit. Nevertheless, these changes were not observed in the objective measurement of performance levels (i.e., PVT). A partial mismatch between subjective and objective assessment of vigilance has been previously reported with sleep curtailment [70]–[72]. Subjective mood levels have been shown to follow a circadian variation, with lower levels in the early morning and to be reduced with the amount of time awake [73], consistent with our observations. Prior studies in simulated night shift work have shown a significant correlation between mood and alertness ratings and circadian phase delays, such that mood and alertness levels increase with greater circadian phase delays [14], [18], [74]. Sleep restriction can also contribute to impaired mood, as a week of sleep restriction to 4–5 hours/night can lead to increased sleepiness and reduced subjective mood levels [11]. These studies are consistent with the higher prevalence of depressed mood in men (odds ratio = 2.05) on a rotating shift schedule compared to day workers [75].

Compared to the adapted group, the non-adapted group showed greater sympathetic dominance (increased LF∶HF ratio and reduced HF power) during both daytime and nighttime sleep, more specifically during wake while in bed, REM sleep, and possibly S2 sleep. A recent study by Chung et al. showed a reduced RR interval, HF power, and LF power as well as an increased LF∶HF ratio during NREM and REM sleep during daytime sleep compared to nighttime sleep in nurses working rotating shifts [59]. The same group also reported greater sympathetic dominance during sleep in permanent night workers compared to day workers [76]. Circadian phase was not measured in these prior studies, but it is reasonable to assume that the nurses were not adapted to their night schedule after only 2 night shifts when the HRV was measured, consistent with the findings for our non-adapted group. During wakefulness, some studies have reported a shift towards sympathetic dominance during night work compared to day work [77], [78], whereas others found no difference [79]–[82]. We and others have reported a significant circadian variation in HRV across circadian phases in humans [83]–[85]. We recently showed that sleep and circadian processes interact to modulate the HRV and that sleeping at adverse circadian phases could lead to acute HRV changes [33], [34]. The peak-to-through changes reported across circadian phases during sleep periods was ∼23% and 37% for the LF∶HF ratio and HF power, respectively [33]. We can estimate that sleeping at different circadian phases could contribute to similar differences between our adaptation groups.

It is also well known that sleeping at adverse circadian phases can lead to sleep restriction, as was observed in the non-adapted group compared to the adapted group (non-adapted: 6.64±0.32 h; adapted: 7.49±0.10 h, Table 1). We recently reported a shift toward sympathetic dominance with sleep restriction [33], which is consistent with the findings of previous studies [86]–[90]. Using an ultradian sleep-wake cycle procedure, which leads to daily sleep restriction, we observed a ∼11%/day increase and a ∼17%/day reduction in the LF∶HF ratio and HF power, respectively [33]. We previously showed that sleep is reduced to ∼5.91 h per 24-h day using a similar procedure in women [91]. In the present study, we can thus estimate that sleep restriction would lead to a ∼77–119% change in HRV after 7 consecutive days of restricted sleep in the non-adapted group. When combined, the direct and indirect (i.e., sleep restriction) effects of circadian misalignment on HRV can account for the 2-fold differences in the LF∶HF ratio observed between our adaptation groups. As we observed HRV variations between the adaptation groups during both visits, we propose that the officers in the non-adapted group suffer from chronic circadian misalignment and sleep restriction with their rotating work schedule. Our results thus suggest that HRV during sleep may be a marker of individual susceptibility to circadian desynchrony in rotating shift workers. Although we did not genotype our participants, our results are consistent with those of Viola et al. [92], who reported that individuals with the longer allele of the circadian clock gene *PER3* polymorphism have greater sympathetic cardiac dominance during NREM sleep compared to individuals with the shorter allele.

HRV measured during wakefulness [93], [94] and sleep [95] decreases with age, and it could be argued that age differences between the adapted and non-adapted groups could explain our HRV differences. Based on the model developed by Koskinen et al. [93], we can predict a 31.8% and 16.9% reduction in HF and LF power, respectively, and a 21.8% increase in the LF∶HF ratio in the non-adapted group (32.6 years) compared to the adapted group (26.4 years). In the present study, the increased LF∶HF ratio during sleep in the non-adapted group compared to the adapted group (104% during wakefulness and 81% during REM sleep, and 109% during S2 sleep) were 3.75- to 5-fold higher than the predicted HRV variation due to age [93]. Moreover, when testing for the effect of age on HRV in our study, it was not found to be significant (p≥0.24).

Light exposure during the night work period was increased in the adapted group, which raises the question whether it could have influenced the HRV. Exposure to light of a higher color temperature (6700 K) in the evening (19:00–02:00 h) was shown to suppress cardiac vagal activity during the following sleep period (02:00–09:00 h), most likely because it led to the suppression of melatonin secretion or SWS [96]. In our study, HRV was recorded during the second sleep period in the laboratory, thus after at least 24 h of dim light (<10 lux) or darkness. To our knowledge, no study has shown such a long-term effect of light on HRV.

### Clinical implications

Shift work has been associated with a number of medical conditions, including cardiovascular diseases, metabolic syndrome, diabetes, and cancer [28], [29], [97]. Circadian misalignment between the sleep/wake cycle and the endogenous circadian pacemaker, which is observed in a majority of shift workers, leads to multiple hormonal and metabolic disturbances that could modulate vulnerability to acute or chronic diseases [97]. We and others have observed changes in the autonomic regulation of the heart in shift workers [33], [59], [76]–[78], [98]–[101]. This is clinically relevant as alterations in HRV have been associated with elevated risk of stroke [102], coronary heart diseases [103], and cardiac events [104] in prospective studies of healthy populations. Altered HRV may potentially alter cardiovascular function with long-term exposure [98]–[100]. Wehrens et al. [98] assessed the HRV in a group of experienced shift workers (mean experience: 8.7 years) and age-matched non-shift workers during baseline sleep, sleep deprivation, and recovery sleep [98]. Submitted to the same protocol, experienced shift workers with similar demographics, circadian phase, posture, and food intake had higher sympathetic dominance and lower endothelial function than controls. Thus, shift work not only leads to acute disruption but persistent alterations in HRV, which is consistent with our findings of reduced HRV during nighttime and daytime sleep in our non-adapted group. The concept of allostasis clearly applies to our group of shift workers. Indeed, while the adapted group shows the expected allostatic changes (i.e., physiological and behavioral intervention promoting a return to homeostasis), the non-adapted group presents signs of allostatic load (i.e., wear and tear) and maybe overload (i.e., wear and tear leading to pathophysiology) that resulted from circadian misalignment and sleep deprivation [105]. The group of non-adapted police officers presented sleep restriction following night shifts and impaired performance levels at night.

Reduced performance, alertness and mood, and increased fatigue and sleepiness associated with working at night raise important financial and safety concerns. Relative to daytime work, night shift work (odds ratio (OR) = 1.92) and rotating shift work (OR = 1.48) have been were associated with an increased risk of work injuries in a representative sample of Canadian workers [24]. According to this study, 11.3% of the 2.7 million injury compensation claims awarded in Canada in 2006 could be attributed to shift work [24]. A meta-analysis of 14 studies revealed that working on a night shift or rotating shift schedule was is associated with a 50–100% increase in accident rate [106]. Using the Oregon workers' compensation dataset of Oregon workers, Horwitz et al. [107] calculated the cost of these additional injuries in hospital employees, and reported a 58.5% increased injury rate in addition to an 8.5% higher cost per claim for night workers compared to day workers. Increased sleepiness after night shifts or extended work shifts has also been associated with an elevated risk of accidents during the commute home period [12], [108], [109], and should not be forgotten as an important public safety issue.

### Conclusion

Circadian adaptation to night shift work is useful to preserve stable mood and performance at night and to improve daytime sleep quality and duration. In this study, circadian misalignment was associated with changes in the autonomic modulation of the heart during sleep. Interestingly, the changes in the SOL and HRV observed in the non-adapted group after the 7 consecutive night shifts were also observed at baseline, suggesting that not only night shifts but also rotating shift work may cause considerable physiological changes in individuals who are susceptible to circadian misalignment and/or sleep restriction. Interventions favoring proper circadian phase entrainment with the work schedule and/or limiting the degree of sleep deprivation appear advantageous to improve physiological and behavioral health in shift workers.

## Supporting Information

Figure S1 Transmittance level of the orange-tinted goggles [see reference 2 of Methods S1].
(TIF)

Figure S2 Relative spectral irradiance of the estimated outdoor light [see reference 1 of Methods S1] and filtered by the orange-tinted goggles [see reference 2 of Methods S1]. When transformed into illuminace [see reference 3 of Methods S1], we estimate that the orange-tinted goggles transmit 48% of the environmental light.
(TIF)

Figure S3 Relative spectral irradiance of the estimated outdoor light and filtered by the orange-tinted goggles, and weighted by the circadian sensitivity as defined in [see reference 4 of Methods S1]. We estimate that the orange-tinted goggles block about 96.7% of the effects of environmental light on the circadian system. The filtered light spectrum was small, so we scaled it up to provide better appreciation of the different size between curves.
(TIF)

Table S1 Time period for light calculation per group.
(DOCX)

Methods S1 Calculation of a transmission coefficient for the orange-tinted goggles.
(DOCX)
