# Supplementary figures and images for: Circadian Adaptation to Night Shift Work Influences Sleep, Performance, Mood and the Autonomic Modulation of the Heart
Source: PLoS One. 2013 Jul 26;8(7):e70813. doi: 10.1371/journal.pone.0070813 (PMC3724779; doi:10.1371/journal.pone.0070813)

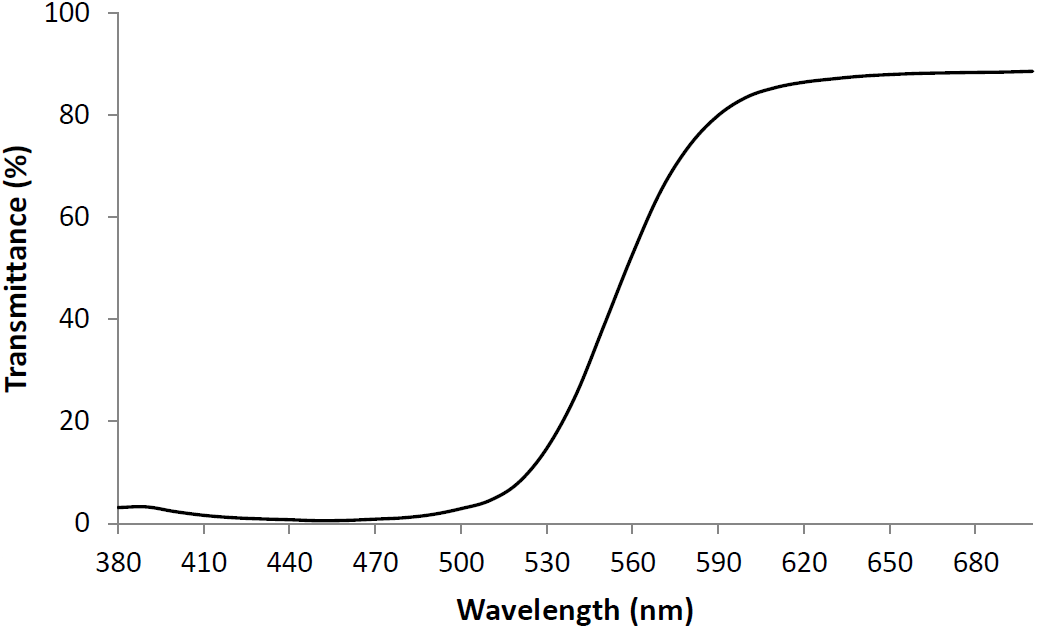

Supplement: Figure S1 — Transmittance level of the orange-tinted goggles [see reference 2 of Methods S1]. (TIF) [file pone.0070813.s001.tif]

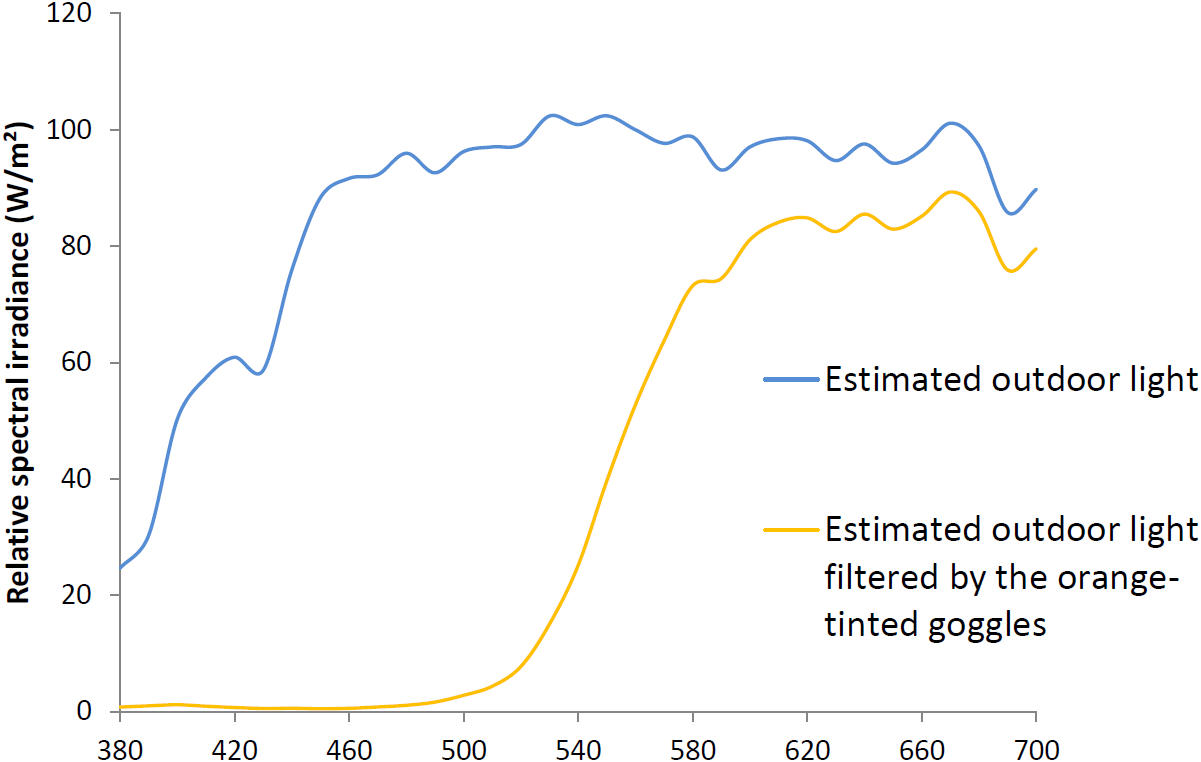

Supplement: Figure S2 — Relative spectral irradiance of the estimated outdoor light [see reference 1 of Methods S1] and filtered by the orange-tinted goggles [see reference 2 of Methods S1]. When transformed into illuminace [see reference 3 of Methods S1], we estimate that the orange-tinted goggles transmit 48% of the environmental light. (TIF) [file pone.0070813.s002.tif]

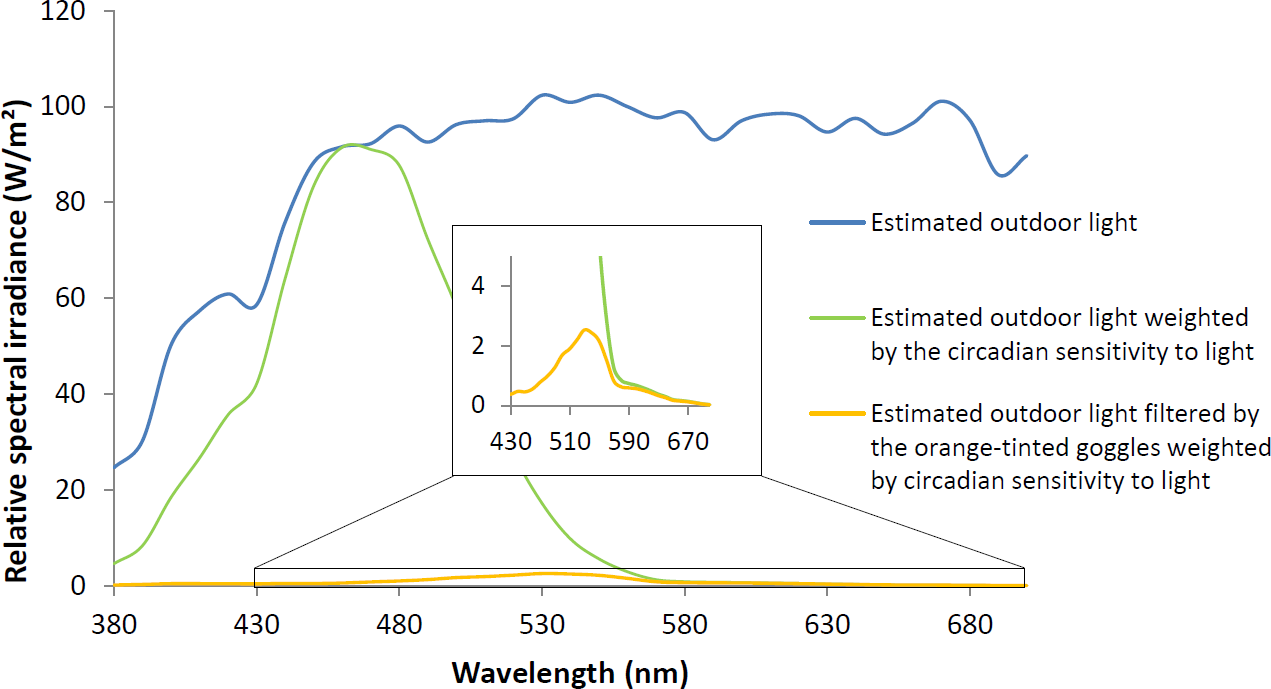

Supplement: Figure S3 — Relative spectral irradiance of the estimated outdoor light and filtered by the orange-tinted goggles, and weighted by the circadian sensitivity as defined in [see reference 4 of Methods S1]. We estimate that the orange-tinted goggles block about 96.7% of the effects of environmental light on the circadian system. The filtered light spectrum was small, so we scaled it up to provide better appreciation of the different size between curves. (TIF) [file pone.0070813.s003.tif]
