# Supplementary material for: Circadian Adaptation to Night Shift Work Influences Sleep, Performance, Mood and the Autonomic Modulation of the Heart
Source: PLoS One. 2013 Jul 26;8(7):e70813. doi: 10.1371/journal.pone.0070813 (PMC3724779; doi:10.1371/journal.pone.0070813)
Supplement: Table S1 — Time period for light calculation per group. (DOCX) [file pone.0070813.s004.docx]

**Table S1.** Time period for light calculation per group

|  | **Average start time** | **Average duration** |
| --- | --- | --- |
| ***Non-adapted group*** |  |  |
| *Night Shift* | 23:07 ± 00:14 | 08:22 ± 00:05 |
| *Morning commute* | 07:30 ± 00:19 | 02:09 ± 00:33 |
| *Daytime sleep* | 09:39 ± 00:40 | 07:28 ± 00:22 |
| *Evening prior to shift* | 17:08 ± 00:24 | 05:59 ± 00:17^t^ |
| ***Adapted group*** |  |  |
| *Night Shift* | 23:05 ± 00:05 | 08:30 ± 00:00 |
| *Morning commute* | 07:35 ± 00:05 | 02:03 ± 00:04 |
| *Daytime sleep* | 09:38 ± 00:05 | 08:13 ± 00:20 |
| *Evening prior to shift* | 17:52 ± 00:19 | 05:12 ± 00:20 |

^t^ indicate a trend (p=0.078) for a difference between adaptation group.
