# Supplementary material for: Circadian Adaptation to Night Shift Work Influences Sleep, Performance, Mood and the Autonomic Modulation of the Heart
Source: PLoS One. 2013 Jul 26;8(7):e70813. doi: 10.1371/journal.pone.0070813 (PMC3724779; doi:10.1371/journal.pone.0070813)
Supplement: Methods S1 — Calculation of a transmission coefficient for the orange-tinted goggles. (DOCX) [file pone.0070813.s005.docx]

**Methods S1**

*Calculation of a transmission coefficient for the orange-tinted goggles.*

The outdoor light spectrum was estimated using the model defined by Judd et al. 1964 [[1](#_ENREF_1)]. More specifically, a color temperature of 5000K was used as input to this model. The resulting estimated morning light irradiance spectrum, between 380-700 nm, is represented in Figure S2 and S3. The transmittance level of the orange-tinted goggles used in this experiment is shown in Figure S1 [[2](#_ENREF_2)]. To correct for the use of the orange-tinted goggles, the estimated outdoor light spectrum was multiplied at different wavelengths (from 380 to 700 nm, using 10 nm steps) by the transmittance level of the orange-tinted goggles (Figure S1) resulting in the filtered outdoor light spectrum (Figure S2). We report illuminance and not irradiance in this study. Thus, the irradiance (W/m^2^) of both filtered and non-filtered estimated outdoor light was transformed into illuminance (lux) using the luminosity function [[3](#_ENREF_3)]. A transmission coefficient was calculated by dividing the relative filtered illuminance by the relative non-filtered illuminance. This calculation resulted in a 48% transmission coefficient. These steps were repeated with different outdoor light spectrums as defined by Judd et al. 1964 [[1](#_ENREF_1)] and resulted in a transmission coefficient ranging from ~46 to 50%.

In a second analysis, we tried estimate the impact of orange-tinted goggles on the effect of light on the circadian system. We thus weighted the spectrum calculated in Figure S2 by the circadian sensitivity to light as reported by Brainard et al. 2001 using melatonin suppression tests under monochromatic light of different wavelengths [[4](#_ENREF_4)]. The resulting relative irradiance spectrum is shown in Figure S3. The radian power (in W) was then calculated for each spectrum, and a transmission coefficient, corrected for the circadian sensitivity to light, was calculated. This transmission coefficient was 3.3% indicating that the orange-tinted goggles block about 96.7% of the resetting effect of environmental light on the circadian system.

**References**

1. Judd DB, MacAdam DL, Wyszecki G, Budde H, Condit H, et al. (1964) Spectral distribution of typical daylight as a function of correlated color temperature. JOSA 54: 1031-1040.

2. Sasseville A, Benhaberou-Brun D, Fontaine C, Charon MC, Hebert M (2009) Wearing blue-blockers in the morning could improve sleep of workers on a permanent night schedule: a pilot study. Chronobiol Int 26: 913-925.

3. Wyszecki G, Stiles WS (2000) Color Science: Concepts and Methods, Quantitative Data and Formulae (2nd eds.): John Wiley & Sons.

4. Brainard GC, Hanifin JP, Greeson JM, Byrne B, Glickman G, et al. (2001) Action spectrum for melatonin regulation in humans: evidence for a novel circadian photoreceptor. J Neurosci 21: 6405-6412.
